# Pesticide Exposure Alters Follicle-Stimulating Hormone Levels in Mexican Agricultural Workers

**DOI:** 10.1289/ehp.7374

**Published:** 2005-05-10

**Authors:** Rogelio Recio, Guadalupe Ocampo-Gómez, Javier Morán-Martínez, Victor Borja-Aburto, Malaquías López-Cervantes, Marisela Uribe, Luisa Torres-Sánchez, Mariano E. Cebrián

**Affiliations:** 1 Sección Externa de Toxicología, Centro de Investigación y de Estudios Avanzados del Instituto Politécnico Nacional, México DF, México; 2 Departamento de Salud Ambiental, Centro de Investigación Biomédica, Facultad de Medicina de Torreón, Universidad Autónoma de Coahuila, Torreón, Coahuila, México; 3 Instituto Mexicano del Seguro Social, México DF, México; 4 Instituto Nacional de Salud Pública, Cuernavaca, Morelos, México.

**Keywords:** alkylphosphates, endocrine disruptors, estradiol, FSH, LH, organophosphorous pesticides, prolactin, testosterone

## Abstract

Organophosphorous pesticides (OPs) are suspected of altering reproductive function by reducing brain acetylcholinesterase activity and monoamine levels, thus impairing hypothalamic and/or pituitary endocrine functions and gonadal processes. Our objective was to evaluate in a longitudinal study the association between OP exposure and serum levels of pituitary and sex hormones. Urinary OP metabolite levels were measured by gas–liquid chromatography, and serum pituitary and sex hormone levels by enzymatic immunoassay and radioimmunoassay in 64 men. A total of 147 urine and blood samples were analyzed for each parameter. More than 80% of the participants had at least one OP metabolite in their urine samples. The most frequent metabolite found was diethylthiophosphate (DETP; 55%), followed by diethylphosphate (DEP; 46%), dimethylthiophosphate (DMTP; 32%), and dimethyldithiophosphate (DMDTP; 31%). However, the metabolites detected at higher concentrations were DMTP, DEP, DMDTP, and dimethylphosphate. There was a high proportion of individuals with follicle-stimulating hormone (FSH) concentrations outside the range of normality (48%). The average FSH serum levels were higher during the heavy pesticide spraying season. However, a multivariate analysis of data collected in all periods showed that serum FSH levels were negatively associated with urinary concentrations of both DMTP and DMDTP, whereas luteinizing hormone (LH) was negatively associated with DMTP. We observed no significant associations between estradiol or testosterone serum levels with OP metabolites. The hormonal disruption in agricultural workers presented here, together with results from experimental animal studies, suggests that OP exposure disrupts the hypothalamic–pituitary endocrine function and also indicates that FSH and LH are the hormones most affected.

Several pesticides have been considered endocrine disruptors because of their capacity to block or activate hormone receptors and/or to affect sex hormone levels (Vinggaard et al. 2000). Epidemiologic studies have suggested associations between OP exposure and long-term effects, such as reproductive disorders (infertility, birth defects, adverse pregnancy outcomes, perinatal mortality) and neurotoxicity (polyneuropathy, neurobehavioral hazards, Parkinson’s disease) (Baldi et al. 1998). Organophosphorous pesticides (OPs) are suspected to alter reproductive function by reducing brain acetylcholinesterase (AChE) activity and secondarily influencing the gonads. Studies on experimental animals have shown that the AChE inhibitor diisopropyl fluorophosphate (DFP), and repeated doses of OP significantly decreased brain AChE activity and significantly increased acetylcholine, gamma-aminobutyric acid, epinephrine, norepinephrine, dopamine, and 5-hydroxytryptamine concentrations (Glisson et al. 1974; Gupta et al. 1984). In addition, organophosphate and carbamate pesticides have been shown to alter pituitary–thyroid and pituitary–adrenal axes and affect prolactin serum levels (Clement 1985; Kokka et al. 1987). OP exposure was also reported to decrease brain AChE activity in mice, which in turn is associated with reduced serum levels of luteinizing hormone (LH) and progesterone and decreased egg production (Rattner and Michael 1985). Smallridge et al. (1991) reported that rats treated with low DFP doses showed increased serum LH levels whereas treatment with higher doses decreased LH and prolactin serum levels. The blunted LH and prolactin response to gonadotropin-releasing hormone (GnRH) induced by DFP led the authors to conclude that the effect was, at least in part, due to a suppressive effect at the pituitary level. More recently, Spassova et al. (2000) reported increased adrenocorticotropic hormone (ACTH) plasma levels and serum corticosterone and aldosterone levels in rats exposed to acephate and metamidophos and suggested that the excess acetylcholine stimulated the release of the hypothalamic corticotropin-releasing hormone, which in turn stimulated ACTH secretion. Sarkar et al. (2000) reported that subchronic oral administration of quinalphos (7–14 mg/kg/day for 15 days) to male rats resulted in increased serum concentrations of LH, follicle-stimulating hormone (FSH), prolactin, and testosterone, without significant effects on dopamine, noradrenaline, or serotonin levels in the hypothalamus or pituitary. Nag and Nandy (2001) reported a significant inhibition of monoamine oxidase-A (MAO-A) and MAO-B, the two main dopamine-degradative enzymes, in rat brain mitochondria exposed to OPs and also that the reversibility of the effect was dependent on the OP used. Similarly, Choudhary et al. (2002) showed that rats treated subcutaneously with the OP dichlorvos presented an increase in dopamine and norepinephrine levels accompanied by increases in the activity of tyrosine and dopamine β-hydroxylases and concomitant decreases in MAO activity, suggesting that the OP-induced decreased prolactin resulted from dopaminergic inhibition, because prolactin secretion is primarily under inhibitory control by dopamine. Thus, the information obtained from these experimental studies suggests that OP exposure alters brain neurotransmitter levels and that the hypothalamic–pituitary axis is a direct target for OP toxicity in rodents.

In contrast, little information is available on the endocrine effects of OP on humans. Güven et al. (1999) reported that, in suicidal individuals suffering from acute OP poisoning, ACTH, prolactin, and cortisol serum levels were significantly higher during poisoning, whereas FSH levels were significantly lower and hormone levels returned to normal after poisoning resolution. Straube et al. (1999) reported a decrease in testosterone and estradiol serum levels in pesticide sprayers 1 day after acute exposure to a wide variety of pesticides; however, chronic exposure resulted in higher LH and testosterone levels in chronically exposed men. In contrast, Padungtod et al. (1998) studied Chinese OP factory workers exposed to ethylparathion and metamidophos and reported that urinary *p*-nitrophenol levels 1 hr after the work shift were positively correlated with serum and urinary FSH levels. Multivariate analysis indicated that OP exposure significantly increased serum LH and FSH levels, whereas testosterone levels decreased. The authors suggested that the abnormal pituitary and sex hormonal patterns found were secondary to testicular damage. In contrast, Larsen et al. (1999) concluded that the use of a wide range of fungicides, insecticides, and herbicides by Danish farmers was not a likely cause of short-term effects on semen quality and reproductive hormones (testosterone, FSH, LH, and inhibin). However, little information is available on the associations between biologic markers of exposure and endocrine effects of OP on agricultural workers. Therefore, our objectives were to evaluate in a longitudinal study the association between OP exposure, as assessed by dialkylphosphate (DAP) urinary excretion, and serum levels of pituitary and sex hormones in Mexican agricultural workers.

## Materials and Methods

### Study population.

We conducted a longitudinal study in the agricultural community of Villa Juarez, State of Durango, Mexico, during 1997–1998. We selected this area because it is surrounded by agricultural fields whose main products are vegetables. Methyl parathion, metamidophos, endosulfan, dimethoate, and diazinon were the pesticides applied most frequently. We randomly selected 230 men from a household sampling frame. Eligible subjects were residents of this community for at least 15 years (average, 24 ± 11.8 years) and had no history of chemotherapy, radiotherapy, or chronic illnesses. One hundred thirty-two (56.4%) healthy men agreed to participate, but only 64 provided a complete set of samples. Each individual was interviewed directly regarding his sociodemographic characteristics, occupational activities, alcohol consumption, smoking habits, and clinical characteristics. Each participant signed an informed consent form and donated blood and urine samples. The study protocol was approved by the Ethics Committee of the School of Medicine, University of Coahuila, Mexico.

### Sample collection.

Samples were collected during the main three periods of the agricultural cycle: crop preparation (November through February) in which small quantities of OP pesticides are regularly sprayed (henceforth called the low-exposure period), heavy spraying season (March through June) when large quantities of pesticides are sprayed (high-exposure period), and/or during the months of July through October in which medium quantities of OP are sprayed (medium-exposure period). A minimum of one and a maximum of five pairs of samples [1 (32.8%), 2 (29.7%), 3 (18.7%), 4 (14.1%), or 5 (4.7%)] were obtained from each subject during the study. Therefore, a total of 147 samples were analyzed for each parameter (51 obtained in the low-exposure period, 53 in the high-exposure period, and 43 in the medium-exposure period). The low- and medium-exposure groups were combined for the bivariate analysis in order to increase statistical power.

### Hormone analysis.

Serum levels of pituitary hormones LH, FSH, and prolactin were measured by enzymatic immunoassay. Estradiol and testosterone were measured by radioimmunoassay. For both assays, we used methods and reagents provided by the World Health Organization (WHO 1999). Three internal and external WHO quality control samples were included in each assay run. Detection limits for LH and FSH were 0.01 IU/mL, and those for prolactin, estradiol, and testosterone were 0.01 ng/mL, 0.06 pg/mL, and 0.01 ng/mL, respectively. The mean intra- and interassay coefficients of variation for all hormones under study were between 8 and 13%. We used the normal ranges of WHO hormone levels as reference values (WHO 1999).

### Urine collection and OP metabolite analysis.

A spot morning urine sample was collected from each participant before blood sample collection and frozen (–70°C) until analysis. We measured OP metabolites dimethylphosphate (DMP), diethylphosphate (DEP), dimethylthiophosphate (DMTP), dimethyldithiophosphate (DMDTP), diethylthiophosphate (DETP), and diethyldithiophosphate (DEDTP) by gas chromatography at Centro de Investigación y de Estudios Avanzados del Instituto Politécnico Nacional according to Aprea et al. (1996). Detection limits for the six metabolites ranged from 1 to 200 ng/mL. The mean intra- and interassay coefficients of variation for OP metabolites under study were between 6 and 11%. Total DAP was calculated as the sum of the six metabolites.

### Statistical analysis.

General characteristics of the study population were described with arithmetic means, SEs, and proportions. Urinary metabolites and serum hormone mean levels were compared among agricultural periods by means of Student’s *t-*, Mann-Whitney, or chi-square tests, depending on the type of variable and its distribution. Dependent variables were transformed to normalize their distribution. Crude associations between individual metabolites and hormone levels were estimated by means of a generalized estimating equation (GEE) to account for the lack of independence of observations (McCullagh and Nelder 1989). Variables that changed the crude regression coefficients by > 10% were considered confounders [age, body mass index (BMI), occupation, smoking, and alcohol intake]. Multivariate adjusted GEE models were further calculated. To account for the multiple comparisons made in our statistical analyses and in an attempt to avoid type 1 statistical errors, we reduced the cutoff point for statistical significance from 0.05 to 0.01. All analyses were performed using STATA statistical software (version 7.0; Stata Corp., College Station, TX, USA).

## Results

### Subjects and urinary DAP levels.

Table 1 shows some characteristics of the population under study. There were no significant differences in relation to age, BMI, occupation, smoking, and alcohol intake between low/medium- and high-exposure groups (data not shown). Regarding DAPs, > 80% of the participants had at least one OP metabolite in their urine samples. The most frequent metabolite found was DETP (55%), followed by DEP (46%), DMTP (32%), and DMDTP (31%). However, the metabolites detected at higher concentrations were DMTP, DEP, DMDTP, and DMP (Table 2). Except for DETP, urinary metabolite levels were higher during the high-exposure period.

### Hormone levels.

A high proportion of individuals (48%) had serum FSH levels outside the range of normality (1.2–5.0 IU/mL) proposed by the WHO (1999). The average FSH serum levels were higher (*p* < 0.05) during the heavy pesticide spraying season (high-exposure period) than during the low- and middle-exposure periods (Table 3). However, a multivariate analysis of data collected in all periods showed that serum FSH levels were negatively associated with both DMTP and DMDTP urinary concentrations (Table 4).

Most studied individuals (~92%) had prolactin serum levels within WHO (1999) normality range (1–19 ng/mL) without significant differences among periods or significant associations with urinary OP metabolites. Regarding LH, 71% of the individuals studied had serum levels within the WHO (1999) normality range (2.5–9.8 IU/L). A multivariate analysis of data collected in the three periods showed that LH serum levels were negatively associated (*p* = 0.008) with DMTP urinary concentrations (Table 4).

Regarding sex hormone levels, most studied individuals (72%) had estradiol serum levels within the WHO (1999) normality range (10–60 pg/mL) without significant differences among periods. We observed no significant associations between estradiol serum levels and urinary excretion of OP metabolites (Table 4). Most individuals (89%) had testosterone serum levels within the WHO (1999) normality range (2.7–9.0 ng/mL) without significant differences among periods. We found no associations between testosterone serum concentration and urinary OP metabolites.

## Discussion

Our main findings in this study were the high proportion of agricultural workers with FSH serum levels outside the range of normality and the inverse relationship between DMTP urinary concentrations and FSH and LH levels. Total DAP urinary levels in nonoccupationally exposed Mexican individuals were twice as high as those reported for the Italian general population (Aprea et al. 1996). The levels in Mexican pesticide sprayers were 190 times higher than those reported for the Italian general population and 6 times higher than those in American greenhouse workers (O’Rourke et al. 2000), suggesting that Mexican individuals living or working in rural environments are highly exposed to OPs. The effects on FSH and LH in the present study were in partial agreement with those reported by Güven et al. (1999) in acutely OP-intoxicated individuals showing significant decreases in serum FSH levels and increases in prolactin without changes in LH levels. In contrast, Padungtod et al. (1998) reported that OP exposure, measured as urinary *p*-nitrophenol levels, significantly increased serum LH and FSH levels, whereas those of testosterone were decreased.

Our results were also in partial agreement with those obtained from studies on experimental animals that suggested the presence of dose-dependent stimulatory and inhibitory effects of OP on the endocrine pituitary function. For example, Smallridge et al. (1991) reported that rats treated with low DFP doses showed increased serum LH levels, whereas treatment with higher doses decreased LH and prolactin serum levels. In contrast, Sarkar et al. (2000) reported that subchronic administration of quinalphos increased serum levels of LH, FSH, prolactin, and testosterone. Despite evidence shown in experimental animals (Sarkar et al. 2000; Smallridge et al. 1991) and humans (Güven et al. 1999), our study did not show significant alterations in prolactin serum levels. The clinical significance of the disruptive effects of OP exposure on FSH and LH homeostasis in the present study could be related to an abnormal gametogenic testicular function.

Regarding effects on steroidal hormones, our findings were not in agreement with studies on OP-exposed individuals reporting decreases in testosterone and estradiol serum levels (Padungtod et al. 1998; Straube et al. 1999), which were in agreement with experimental studies reporting OP-induced alterations in testosterone and estradiol metabolism (Butler and Murray 1993; Murray and Butler 1995). Nonetheless, there is a need for further studies measuring urinary and/or serum concentrations of sexual hormones and their metabolites in human exposed populations. In addition to the dose-dependent stimulatory or inhibitory effects of OP on endocrine function mentioned above, the lack of consistency among studies assessing the effects of pesticide exposure on human hormone levels could also be due to differences in length and severity of exposures, protection equipment patterns of use, agrochemicals used, and/or agricultural practices, which play an important role in determining the characteristics of endocrine effects. Regarding the relationship between OP metabolites and endocrine effects, it is likely that urinary levels of OP metabolites reflect the magnitude of the exposure. However, further experimental studies are required to establish whether OP metabolites are able to induce alterations in the neuroendocrine system, notwithstanding that OP metabolites are assumed to have fewer biologic effects than do their parent compounds or their highly reactive oxons.

In summary, the hormonal disruption in agricultural workers in the present study, together with results from experimental animal studies, suggests that OP exposure disrupts the hypothalamic–pituitary endocrine function and also indicates that FSH and LH are the hormones most affected.

## Figures and Tables

**Table 1:** Selected characteristics of the study population (*n* = 64).

Characteristic	Prevalence^a^
Age (years)	
Mean ± SD (range)	28.6 ± 8.8 (18–50)
Occupation	
Agricultural workers	25.4
Pesticide sprayers	19.0
Other	55.6
Body mass index (kg/m^2^)	
Mean ± SD (range)	26.1 ± 4.2 (20.2–42.2)
Tobacco smoking (no. of cigarettes/day)	
None	40.6
1–4	31.3
5–9	10.9
≥10	17.2
Alcohol intake (glasses of wine/month)	
0–6	28.1
7–21	25.0
24–36	29.7
42–60	17.2

a Values are percent except where noted.

**Table 2:** Mean ± SD (range) of urinary OP metabolite levels (ppb) by agricultural period.

	Period of exposure	
Metabolite	Low/medium (*n* = 104)^a^	High (*n* = 43)^a^
DMP	56.2 ± 249.7 (ND–2169.1)	62.5 ± 169.1 (ND–994.1)
DMTP	694.6 ± 1531.3 (ND–7383.0)	3790.8 ± 19183.9 (ND–124441)
DMDTP	101.8 ± 273.4 (ND–2148.6)	333.4 ± 889.5 (ND–3971.3)
DEP	150.4 ± 630.4 (ND–4144.3)	217.1 ± 743.6 (ND–3492.3)
DETP	43.4 ± 54.5 (ND–394.3)	32.1 ± 56.1* (ND–202.1)
DEDTP	33.8 ± 153.0 (ND– 1062.0)	94.4 ± 251.1 (ND–938.1)
Total DAP	1170.3 ± 2318.3 (ND–11525.6)	4406.5 ± 19885.4 (ND–130430.7)

ND, nondetectable,

a Number of samples.

* p < 0.05 by Mann-Whitney’s test.

**Table 3:** Mean ± SD (range) of serum hormone levels by agricultural period.

	Period of exposure	
Hormone	Low/medium (*n* = 104)^a^	High (*n* = 43)^a^
LH (IU/L)	4.2 ± 2.7 (0.2–15.2)	4.8 ± 2.8 (0.1–10.3)
FSH (IU/L)	4.8 ± 2.7 (0.3–13.9)	6.1 ± 2.9* (0.5–19.1)
Prolactin (ng/mL)	7.5 ± 5.1 (0.2–25)	6.7 ± 4.3 (0.2–16.5)
Estradiol (pg/mL)	17.4 ± 11.9 (0.1–55.7)	20.2 ± 12.7 (1.8–46.0)
Testosterone (ng/mL)	4.5 ± 1.6 (1.3–9.6)	4.8 ± 2.0 (1.9–12.3)

a Number of samples.

* p < 0.05 by t-test on transformed variable.

**Table 4:** Associations between serum hormone levels and urinary OP metabolite concentrations (*n* = 147 samples).

	LH (IU/mL)		FSH (IU/mL)		Prolactin (ng/mL)		Estradiol (pg/mL)	
Metabolite	β(SE)	*p*-Value	β (SE)	*p*-Value	β (SE)	*p*-Value	β (SE)	*p*-Value
DMP	–0.21^a^ (0.55)	0.53	0.03^b^ (0.6)	0.83	–0.66^c^ (1.0)	0.42	–13.05^d^ (7.6)	0.19
DMTP	–0.0002 (0.0003)	0.008	–0.002^e^ (0.0003)	0.007	–0.00004^f^ (0.0005)	0.78	–0.018^d^ (0.06)	0.60
DMDTP	–0.24^e^ (0.11)	0.13	–1.0 (0.09)	0.001	–0.02^g^ (0.20)	0.77	–1.40^h^ (2.34)	0.44
DEP	0.20 (0.06)	0.08	–0.15^i^ (0.06)	0.11	–0.02^c^ (0.12)	0.69	–1.59^a^ (0.37)	0.04
DETP	0.0009^g^ (10.1)	0.99	0.08^g^ (9.4)	0.93	0.008^g^ (17.9)	0.98	14.5^j^ (58.6)	0.62
DEDTP	–1.2^e^ (0.8)	0.22	–0.085^g^ (0.8)	0.74	–0.0005^g^ (1.49)	0.99	–0.02^k^ (5.78)	0.95

a Adjusted for occupation.

b Adjusted for age and smoking.

c Adjusted for age and group.

d Adjusted for alcohol intake and occupation.

e Adjusted for BMI.

f Adjusted for age, BMI, alcohol intake, and occupation.

g Adjusted for age, BMI, occupation, smoking, and alcohol intake.

h Adjusted for smoking, alcohol intake, and occupation.

i Adjusted for age.

j Adjusted for smoking and alcohol intake.

k Adjusted for BMI, occupation, smoking, and alcohol intake.
